# Slaves of the Cooking Stoves

**Published:** 1883-04

**Authors:** 


### SLAVES OF THE COOKING STOVE.

WHEN people of the old world begin to mingle freely with Americans, the first thing to attract their attention, usually, is the strange food on the tables. Many articles they never before saw or heard of. But what excites their surprise is the extraordinary number of things provided for each meal.

"Here are seven kinds of cake, *nothing to eat*," we heard a traveler remark one evening at a hotel in Vermont, when he came in hungry from fishing on Lake Champlain.

That's just it. Seven kinds of cake and nothing to eat! He might have added, three kinds of preserves, a jar of mixed pickles and a pile of flapjacks. All this, and nothing to eat! A man of sound digestion and healthy appetite would naturally wave these frivolous dainties aside, and ask for some proper human food. Good bread and butter would answer his purpose. Add baked potatoes, and he would rise from the table refreshed and satisfied, and sleep his allowance of eight hours as unlike the proverbial "top" as possible. What can a hungry man do with pound-cake and pickles?

But, ladies, of all the viands ordinarily seen on tables, this trash is the most laborious to prepare. It is the eternal round of pie, cake and sweetmeats that wears out so many noble women in the country, who would rather die than come short of what they think is their duty to their households.

The remedy for this is a more rational mode of cookery. Why spoil good fruit by flattenimg it out into innutritious pie? Good bread, good meat, good vegetables, good fruit—what do we want more? A Scotch farmer gets a good breakfast from oatmeal and milk, and goes to bed on bread and cheese. Ladies, abolish the seven kinds of cake, put on the table something to eat, and let the simpletons growl if they will.—*N. Y. Ledger.*

---

HORSES often suffer from irritation of the skin under the hair of the mane and tail. It may be due to want of proper cleaning of the skin, or to some irritation of the blood caused by dry feed. The parts should be washed with warm water and soap, and then rubbed with alcohol, to which a few drops of tincture of cantharides are added. Give the horse some scalded bran with salt in it for a few days.—*N. Y. Times.*

